# The relationship between sleep habits, lifestyle factors, and achieving guideline-recommended physical activity levels in ten-to-fourteen-year-old Japanese children: A cross-sectional study

**DOI:** 10.1371/journal.pone.0242517

**Published:** 2020-11-13

**Authors:** Takumi Aoki, Kazuhiko Fukuda, Chiaki Tanaka, Yasuko Kamikawa, Nobuhiro Tsuji, Ryoji Kasanami, Taketaka Hara, Ryo Miyazaki, Hideki Tanaka, Hidenori Asai, Naofumi Yamamoto, Kan Oishi, Kojiro Ishii

**Affiliations:** 1 Graduate School of Health and Sports Science, Doshisha University, Kyoto, Japan; 2 Japan Society for the Promotion of Science, Tokyo, Japan; 3 College of Sociology, Edogawa University, Chiba, Japan; 4 College of Health and Welfare, J. F. Oberlin University, Tokyo, Japan; 5 Advisor, Toyama University, Toyama, Japan; 6 Graduate School of Education, Shiga University, Shiga, Japan; 7 Faculty of Education, Nara University of Education, Nara, Japan; 8 Faculty of Education, Shimane University, Shimane, Japan; 9 Faculty of Human Sciences, Shimane University, Shimane, Japan; 10 Faculty of Psychology, Hiroshima International University, Hiroshima, Japan; 11 Faculty of Collaborative Regional Innovation, Ehime University, Ehime, Japan; 12 Faculty of Health and Sports Science, Doshisha University, Kyoto, Japan; La Inmaculada Teacher Training Centre (University of Granada), SPAIN

## Abstract

The current focus of meeting the physical activity guidelines for children and young people include preventing conditions such as high blood cholesterol, high blood pressure, metabolic syndrome, obesity, low bone density, depression, and injuries. However, the relationship between sleep habits and meeting physical activity guidelines is still unclear. This study aimed to assess this relationship among fifth- to eighth-grade (ages 10–14) Japanese children. This cross-sectional study included 3,123 children (boys: 1,558, girls: 1,565, mean age: 12.5 ± 1.2 years). Questionnaires were used to assess parameters such as moderate-to-vigorous physical activity per day, school and weekend night sleep durations, social jetlag, daytime sleepiness, napping, screen time, and breakfast intake. Participants were divided into an achievement and a non-achievement group depending on their physical activity guideline achievement status (i.e., whether they met the children’s physical activity guideline of 60 min or more of moderate-to-vigorous physical activity per day). Then, to determine the sleep habits in relation to the children’s achievement of guideline-recommended physical activity levels, multivariate logistic regression analyses were conducted. In fifth- and sixth-grade (ages 10–12) boys, an inverse association was observed between physical activity guideline achievement and daytime sleepiness. In seventh- and eighth-grade (ages 12–14) boys, physical activity guideline achievement was inversely associated with social jetlag and skipping breakfast. Additionally, in seventh- and eighth-grade girls, physical activity guideline achievement was inversely associated with inappropriate sleep duration on weekends and screen time. These results suggest that meeting the physical activity guideline is related to favorable sleep habits in Japanese children. However, their relevance may differ by school type and gender.

## Introduction

Adolescent children have many sleep-related issues. Gradisar et al. performed a meta-analysis of adolescent sleep patterns and showed that sleep patterns tended to delay with increasing age and sleep duration was reduced accordingly [1]. Furthermore, sleep duration for those in adolescence has decreased by approximately 30 min in the past few decades [2]. In addition, disruptions in sleep habits, such as a shortened sleep duration and an irregular sleep-wake rhythm, are associated with multiple problems, such as increased daytime sleepiness [3], increased obesity risk [4, 5], mental health deterioration [6, 7], and a decline in academic performance [8]. Thus, good sleep habits are extremely important in maintaining and promoting health in adolescent children.

Physical activity is one approach to resolve sleep problems in adolescent children. For example, in their systematic review, Lang et al. noted an association between abundant physical activity and good night-time sleep [9]. Additionally, in their meta-analysis review, Bartel et al. reported that physical activities can become a protective factor for bedtime [10]; thus, physical activity is essential for good sleep health in adolescent children.

Current physical activity guidelines targeting children recommend at least 60 min of physical activity per day [11–13]. However, the outcomes for such activities are focused on preventing ailments such as high blood cholesterol, high blood pressure, metabolic syndrome, obesity, low bone density, depression, and injuries, but there are no items concerning sleep. Thus, the relationship between achieving guideline-recommended physical activity levels and sleep habits is unclear.

As mentioned earlier, multiple reviews [9, 10] have reported a positive association between ample physical activity and good sleep in adolescent children. Therefore, we hypothesized that meeting the guideline-recommended levels of physical activity could also be associated with good sleep habits in children. Verification of this research hypothesis could contribute to the maintenance and improvement of sleep habits and promote public awareness of these physical activity guidelines. However, the published literature contains only a few reports (i.e., Olds et al. [4]) and, more specifically, no such study has been conducted in Japan. Therefore, the purpose of this study was to elucidate the sleep habits of children in relation to the degree to which they met the recommendations of physical activity guidelines.

## Methods

### Research location and participants

This survey was conducted across several regions (11 elementary schools and 10 junior high schools in 1 metropolitan prefecture, 1 urban prefecture, and 8 prefectures proper) in Japan. Even amongst adolescent children, changes in sleep patterns are particularly notable in children in early adolescence. A survey in Japanese children [14] in the higher grades of elementary and junior high school demonstrated sleep duration reductions of more than 1 hour. Therefore, in this study, we conducted our survey during periods of no school events or examinations with a total of 3,778 children in the fifth and sixth grades (ages 10–12) of elementary school and those in the seventh and eighth grades (ages 12–14) of junior high school. Prior to the survey, the research purpose and methods were explained in writing to the principals of the schools. Furthermore, we provided a written explanation of the study to the participants and their parent or guardian and determined that submission of their survey response would be considered consent. This study was approved by the Ethics Committee of Doshisha University (Approval Number: 17095) and conducted in accordance with the Declaration of Helsinki.

### Measurement items for basic information

A questionnaire form was used to collect data pertaining to the grade (school year), gender, height, and weight of the children. Subsequently, the percentile of body mass index was calculated [15].

### Measurement items for physical activity

We used the modified Short Version of the International Physical Activity Questionnaire (Japanese version) [16], the International Physical Activity Questionnaire for Japanese Early Adolescents [17]. Using prior studies as a reference [18], the time spent on moderate-intensity and vigorous-intensity physical activities over the past week was divided by 7 days to calculate the average moderate-to-vigorous physical activity (MVPA) time per day. Next, based on the standards set by the physical activity guidelines (60 min or more MVPA per day) [11–13], participants whose average daily MVPA time was less than 60 min were categorized in the *Non-Achievement Group*, while those whose average daily MVPA time was equal to or more than 60 min a day were categorized in the *Achievement Group*.

### Measurement items for sleep

School and weekend night bedtimes, school day and weekend wake-up times, and school and weekend night sleep durations were each surveyed using a questionnaire. Based on the replies for school and weekend night bedtimes and wake-up times, the midpoint of school night sleep duration (mid-sleep time on work/school days: MSW), as well as the midpoint of the weekend night sleep duration (mid-sleep time on free days: MSF) were each calculated and used in the following formula to determine social jetlag [19].

Socialjetlag=|MSF‐MSW|

To measure daytime sleepiness, the Japanese version of the Pediatric Daytime Sleepiness Scale (PDSS-J) [20] was used. PDSS-J is a self-assessment of the level of sleepiness in eight specific situations that tend to induce sleepiness, with higher scores indicating a greater daytime sleepiness. Additionally, participants were asked to answer questions concerning any naps they take between the time they return from school until their bedtime. Participants were classified into those with 0 min of nap time after school and those with any amount of nap time.

### Other measurement items

Each child responded to questions regarding (1) the time spent watching television, videos, or DVDs; (2) time spent playing video games; and (3) time spent on cell phones, smartphones, tablets, and personal computers, and we calculated the total time spent on (1) to (3) as their screen time per day. The participants also responded to questions on how many times they had breakfast per week to investigate their breakfast intake. Participants were classified into those who had breakfast 7 times per week and those who skipped breakfast.

### Statistical analysis

Participants were stratified by school type (elementary or junior high school) and gender to conduct statistical analyses. The statistical software used in the analyses was SPSS Statistics for Windows 26.0 (IBM Corp., Armonk, NY), and the statistical significance level was set to < 0.05. To compare different school types and genders, two-way analyses of variance were conducted. Furthermore, we used the χ^2^ test on categorical data, and when significant differences were found, a residual analysis was performed.

To determine sleep habits in relation to the children’s achievement of guideline-recommended physical activity levels, we conducted multivariate logistic regression analyses by setting the objective variable as the achievement status of recommended physical activity levels (0: *Non-Achievement Group*, 1: *Achievement Group*) and the explanatory variables as the grade, body mass index percentile, school and weekend night sleep duration, social jetlag, daytime sleepiness, the presence or lack of an after-school nap, screen time, and whether breakfast was skipped. Odds ratio [95% confidence interval] of each explanatory variable was calculated through multivariate logistic regression analyses.

Before conducting multivariate logistic regression analyses, criteria for the school and weekend night sleep duration categories were decided based on the guidelines from the American Academy of Sleep Medicine [21]. Accordingly, for participants in the fifth and sixth grades of elementary school, a sleep duration of 9–12 hours was categorized as *Appropriate*, less than 9 hours was categorized as *< Appropriate*, and more than 12 hours was categorized as *> Appropriate*. For participants in the seventh and eighth grades of junior high school, a sleep duration of 8–10 hours was categorized as *Appropriate*, less than 8 hours was categorized as *< Appropriate*, and more than 10 hours was categorized as *> Appropriate*.

For social jetlag, two categories were created: *< 60 min* and ≥ *60 min* [19]. For PDSS-J scores, two categories were created: *< 17 points* and ≥ *17 points* [22]. Additionally, screen time was divided into quarters and categorized as Q1, Q2, Q3, and Q4.

## Results

### Physical activity-related characteristics

**Fig 1:**
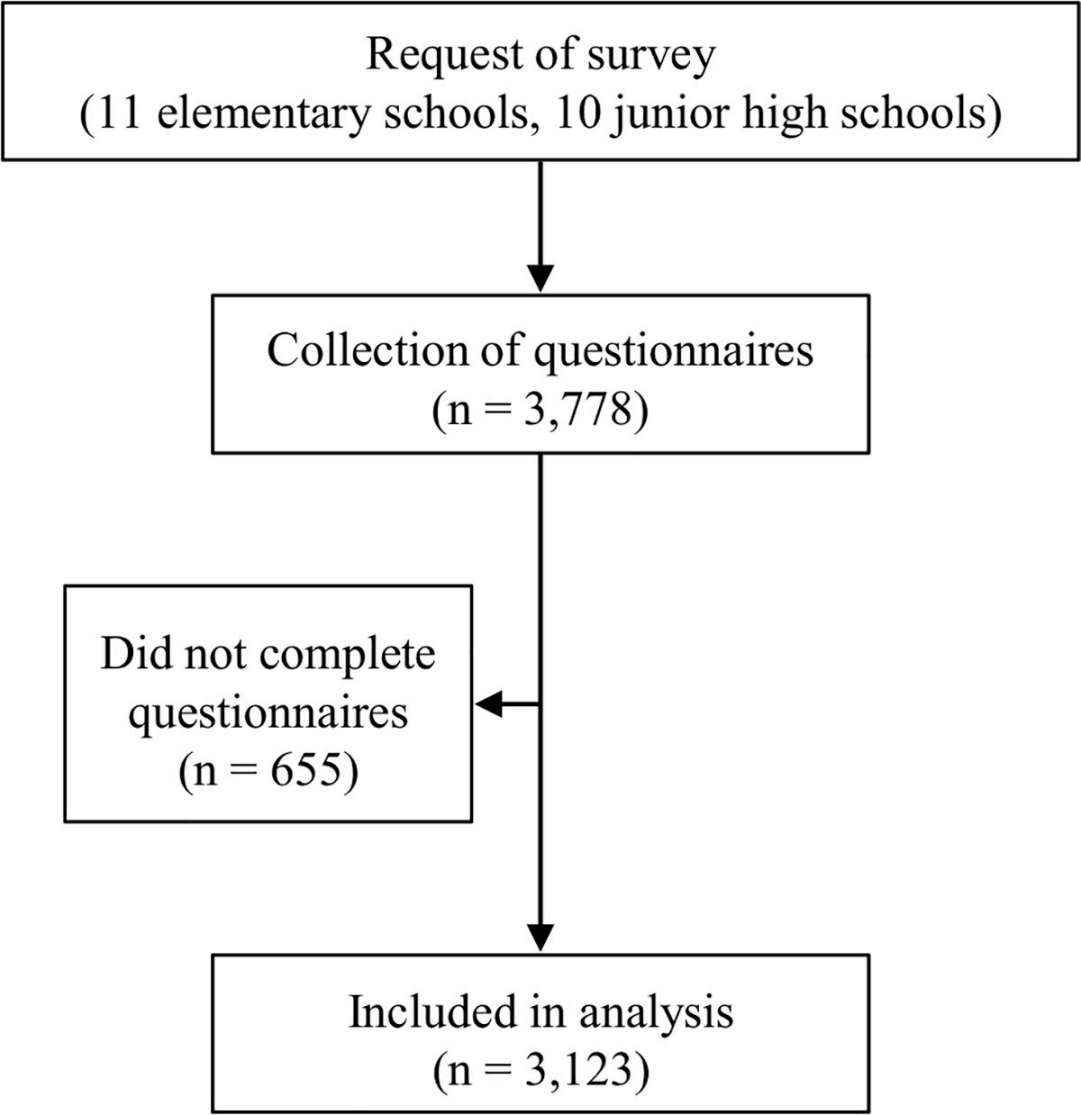
Protocol for research participant enrollment.

A total of 3,123 of the 3,778 children with no missing information in their replies to the questionnaire were included in the analyses (boys: 1,558, girls: 1,565, mean age: 12.5 ± 1.2 years) (effective response rate: 82.7%) (Fig 1). For the rate of achievement of guideline-recommended physical activity levels, the χ^2^ test showed a significant difference based on the type of school and gender of the children (Table 1). Residual analysis showed that boys and girls in the fifth and sixth grades of elementary school had lower achievement rates, while boys in their seventh and eighth grade of junior high school had higher achievement rates (Table 1).

**Table 1:** The characteristics of the research participants.

	School type				Two-way analysis of variance			Chi-square test
	Elementary school students in the fifth & sixth grades		Junior high school students in the seventh & eighth grades		Factor	Factor	Interaction	
	(n = 1,348)		(n = 1,775)		School type	Gender	(School type × Gender)	
	Boys (n = 672)	Girls (n = 676)	Boys (n = 886)	Girls (n = 889)	F	F	F	χ2
Height (cm)	142.9 ± 7.5	144.1 ± 7.4	158.5 ± 8.5	154.4 ± 5.7	2388.24 ***	28.48 ***	101.23 ***	-
Weight (kg)	35.7 ± 7.0	35.9 ± 6.8	46.3 ± 9.4	43.8 ± 7.0	1094.72 ***	19.08 ***	23.36 ***	-
BMI percentile	41.4 ± 27.2	37.3 ± 26.6	36.6 ± 26.3	32.3 ± 25.1	26.91 ***	19.94 ***	0.02	-
Achievement status of physical activity guideline								
Not achieved	51.9% (349)	69.8% (472)	28.3% (251)	48.7% (433)	-	-	-	270.54 ***
Achieved	48.1% (323)	30.2% (204)	71.7% (635)	51.3% (456)	-	-	-	
School night sleep duration (min / day)	486.8 ± 62.5	475.1 ± 66.6	431.3 ± 62.3	414.5 ± 61.9	646.40 ***	39.08 ***	1.21	-
Weekend night sleep duration (min / day)	518.9 ± 86.9	539.8 ± 87.3	493.2 ± 96.3	505.3 ± 91.0	83.82 ***	25.24 ***	1.81	-
Social jetlag (min)	53.5 ± 48.3	64.4 ± 45.7	63.9 ± 57.9	71.3 ± 53.1	21.26 ***	23.88 ***	0.85	-
PDSS-J score (points)	11.2 ± 6.2	12.7 ± 6.1	13.6 ± 6.4	15.8 ± 5.9	159.16 ***	71.42 ***	2.49	-
After school napping								
No	78.3% (526)	72.9% (493)	69.9% (619)	63.1% (561)	-	-	-	44.90 ***
Yes	21.7% (146)	27.1% (183)	30.1% (267)	36.9% (328)	-	-	-	
Screen time (min / day)	250.4 ± 179.7	230.0 ± 168.7	254.5 ± 181.9	239.8 ± 179.5	1.17	7.40 **	0.20	-
Skipped breakfast								
No	89.1% (599)	88.9% (601)	86.9% (770)	83.8% (745)	-	-	-	12.86 **
Yes	10.9% (73)	11.1% (75)	13.1% (116)	16.2% (144)	-	-	-	

Data presented as mean ± standard deviation or % (n).

BMI, body mass index; PDSS-J, Japanese version of the pediatric daytime sleepiness scale

**: p < 0.01.

***: p < 0.001.

### Sleep habit-related characteristics

For school and weekend night sleep durations, social jetlag, and PDSS-J score, main effects were observed in relation to school types and genders (Table 1). Specifically, compared with elementary school students in fifth and sixth grade, junior high school students had irregular sleep habits (Table 1). Likewise, compared to boys, girls had more irregular sleep habits (Table 1). With respect to taking naps, a significant difference was observed via the χ^2^ test (Table 1). Residual analysis showed that boys in the fifth and sixth grades took naps less often while girls in the seventh and eighth grades of junior high school took naps more frequently (Table 1).

### Characteristics related to other factors

With regard to screen time, girls had less screen time than boys (Table 1). With respect to skipping breakfast, a significant difference was observed using the χ^2^ test (Table 1). Residual analysis showed that girls in the seventh and eighth grades of junior high school had a higher rate of skipping breakfast (Table 1).

### Sleep habits related to the recommended levels of physical activity

Regarding the PDSS-J score in elementary school boys in fifth and sixth grade, those with scores equal to or higher than 17 points had a significantly lower odds ratio (0.65 [0.43–0.98]) than those with scores lower than 17 (Table 2). No significant associations for any of the items were found for elementary school girls in the fifth and sixth grades (Table 2).

**Table 2:** Sleep habits relating to physical activity in elementary school students (fifth and sixth grades).

	Boys (n = 672)		Girls (n = 676)	
	OR	95% CI	OR	95% CI
Grade				
Fifth grade	Ref		Ref	
Sixth grade	0.95	0.70–1.30	1.31	0.93–1.86
BMI percentile	1.00	0.99–1.00	0.99	0.99–1.00
School night sleep duration				
Appropriate ^a^	Ref		Ref	
< Appropriate ^b^	1.05	0.70–1.59	0.97	0.58–1.65
Weekend night sleep duration				
Appropriate ^a^	Ref		Ref	
< Appropriate ^b^	1.09	0.77–1.54	1.39	0.97–1.99
> Appropriate ^c^	0.28	0.03–2.56	2.82	0.80–9.92
Social jetlag				
< 60 min	Ref		Ref	
≥ 60 min	1.12	0.80–1.57	0.74	0.51–1.07
PDSS-J score				
< 17 points	Ref		Ref	
≥ 17 points	0.65	0.43–0.98	0.94	0.63–1.41
After school napping				
No	Ref		Ref	
Yes	1.19	0.81–1.74	1.12	0.76–1.64
Screen time				
Q1	Ref		Ref	
Q2	1.33	0.85–2.08	1.56	0.94–2.58
Q3	1.08	0.68–1.73	0.97	0.60–1.58
Q4	1.02	0.63–1.64	1.26	0.78–2.04
Skipped breakfast				
No	Ref		Ref	
Yes	1.06	0.64–1.77	0.93	0.53–1.63

OR, odds ratio; CI, confidence interval; Ref, reference category; BMI, body mass index; PDSS-J, Japanese version of the pediatric daytime sleepiness scale.

^a^ Appropriate: 9–12 hours

^b^ < Appropriate: < 9 hours

^c^ > Appropriate: > 12 hours.

Regarding social jetlag in junior high school boys in their seventh and eighth grade, those with social jetlag equal to or more than 60 min had significantly lower odds ratios (0.57 [0.42–0.79]) than those with less than 60 min (Table 3). Likewise, in the case of those skipping breakfast, a significantly lower odds ratio was indicated (0.58 [0.38–0.88]) compared to the ratio of those not skipping breakfast (Table 3).

**Table 3:** Sleep habits relating to physical activity in junior high school students (seventh and eighth grades).

	Boys (n = 886)		Girls (n = 889)	
	OR	95% CI	OR	95% CI
Grade				
Seventh grade	Ref		Ref	
Eighth grade	1.04	0.76–1.42	1.08	0.82–1.43
BMI percentile	1.00	0.99–1.00	1.00	0.99–1.00
School night sleep duration				
Appropriate ^a^	Ref		Ref	
< Appropriate ^b^	1.00	0.68–1.48	0.75	0.49–1.14
Weekend night sleep duration				
Appropriate ^a^	Ref		Ref	
< Appropriate ^b^	1.26	0.91–1.76	1.17	0.87–1.59
> Appropriate ^c^	1.12	0.66–1.90	0.60	0.37–0.98
Social jetlag				
< 60 min	Ref		Ref	
≥ 60 min	0.57	0.42–0.79	1.06	0.79–1.42
PDSS-J score				
< 17 points	Ref		Ref	
≥ 17 points	1.00	0.72–1.39	1.14	0.86–1.51
After school napping				
No	Ref		Ref	
Yes	0.94	0.68–1.30	1.25	0.94–1.67
Screen time				
Q1	Ref		Ref	
Q2	1.18	0.75–1.87	0.78	0.53–1.14
Q3	1.26	0.80–1.99	0.75	0.50–1.11
Q4	0.99	0.63–1.56	0.64	0.43–0.97
Skipped breakfast				
No	Ref		Ref	
Yes	0.58	0.38–0.88	1.10	0.75–1.60

OR, odds ratio; CI, confidence interval; Ref, reference category; BMI, body mass index; PDSS-J, Japanese version of the pediatric daytime sleepiness scale.

^a^ Appropriate: 8–10 hours

^b^ < Appropriate: < 8 hours

^c^ > Appropriate: > 10 hours.

Regarding sleep duration during weekend nights among junior high school girls in their seventh and eighth grades, girls in the *> Appropriate* (more than 10 hours) category had a significantly lower odds ratio (0.60 [0.37–0.98]) than in the *Appropriate* (8–10 hours) category (Table 3). Likewise, participants in the Q4 (the longest screen time) category had a significantly lower odds ratio (0.64 [0.43–0.97]) than those in the Q1 (the shortest screen time) category (Table 3).

## Discussion

The purpose of the present study was to elucidate the sleeping habits of Japanese adolescent children in relation to their ability to meet the recommended physical activity guidelines. This study revealed that (1) elementary school boys in the fifth and sixth grades showed an inverse correlation between the degree of daytime sleepiness and achievement of guideline-recommended physical activity levels; (2) among students in the seventh and eighth grades of junior high school, achievement of guideline-recommended physical activity levels inversely correlated with a substantial amount of social jetlag and skipped breakfast in boys, as well as sleep duration beyond the recommended amount on the weekends and prolonged screen time in girls.

With respect to the relationship between the amount of physical activity and sleep, prior research concerning fifth-grade elementary school students showed a relationship between lower amounts of physical activity and the magnitude of daytime sleepiness [23]. Additionally, Brand et al. reported in a study concerning youths that students who engaged in frequent physical activity on a daily basis showed good sleep and psychological functioning [24]. The findings of our study support these conclusions; thus, it can be considered that ample physical activity, achieving the guideline-recommended levels, is likely associated with good sleep habits.

In this study, we found a relationship between daytime sleepiness and the achievement of guideline-recommended physical activity levels in boys in the fifth and sixth grades of elementary school; however, for boys and girls in their seventh and eighth grades of junior high school, achieving guideline-recommended physical activity levels was associated with sleep variables concerning the sleep-wake rhythm, such as social jetlag and sleep duration. Hence, the sleep variables associated with achieving guideline-recommended physical activity levels differed based on school type, and/or age. A possible explanation for this finding could be the different opportunities to participate in a sports-related extracurricular school club based on school type. Cave stated that extracurricular school clubs are a characteristic of Japanese schools and become available after middle school (junior high school and beyond), while the frequency of such activities ranges from 6–7 days a week [25]. Thus, we estimate that study participants in the seventh and eighth grades of junior high school maintained their sleep-wake rhythm during school days and weekends due to their affiliation to and engagement in activities of sports-related extracurricular school clubs. Furthermore, we suspect that among these individuals, apart from the physical activity itself being associated with sleep habits, the impact of belonging to and engaging in weekend activities in sports-related extracurricular school clubs may also be associated with the sleep variables concerning sleep-wake rhythm.

There was no clear association between the achievement of guideline-recommended physical activity levels and sleep habits in fifth- and sixth-grade elementary school girls. The rate of achievement of guideline-recommended physical activity levels was 48.1% for boys and 30.2% for girls in the fifth and sixth grades of elementary school, and 71.7% for boys and 51.3% for girls in the seventh and eighth grades of junior high school. Thus, elementary school girls showed the lowest rate of achievement. Previous research has also indicated that only about 30.6% of girls in the fifth grade of Japanese elementary schools engage in physical activity for 420 min a week or more (an average of 60 min or more per day) [26]. Furthermore, it is rare for girls in the fifth and sixth grades of elementary school to achieve the guideline-recommended amount of physical activity. It is plausible that the intergroup differences seen in the present study are due to the low achievement rate among girls in the fifth and sixth grades of elementary school. However, studies in other countries have reported no clear relationship between grade and physical activity level [27], and that physical activity level declines according to grade [28]. Therefore, the low rate of achievement of guideline-recommended physical activity levels in fifth- and sixth-grade elementary school girls may be a phenomenon specific to Japanese children. Further research is needed to examine these differences between countries. In terms of strategies to increase the amount of physical activity, Trott et al. reported that participants who performed physical activity in groups gained higher levels of enjoyment and more total steps than those who performed physical activity alone [29]. As such, encouraging physical activity in groups is expected to increase the amount of physical activity and improve the rate of achievement of guideline-recommended physical activity levels.

In terms of skipping breakfast, findings have been inconsistent, with some studies reporting that it is associated with physical activity [30], while others have reported that they are unrelated [31]. However, in the present study, the rate of achieving the guideline-recommended physical activity level was related to skipping of breakfast in seventh and eighth grade boys of junior high school. Additionally, among light exposure [32] and physical activities [33], meals may potentially act as an entrainment factor in circadian rhythms [34], at least from the perspective of achieving the guideline-recommended physical activity levels. Therefore, it might be necessary to consider and address eating habits as well.

Screen time is an indicator of sedentary behavior. A meta-analysis conducted by Pearson et al. indicated an inverse association between sedentary behavior and physical activity [35]; hence, the duration of sedentary behavior arising from screen time may be a factor explaining the inverse association between achieving guideline-recommended physical activity levels in girls in the seventh and eighth grades of junior high school. However, sedentary behavior and physical activity are necessarily not antithetical to one another [35]. To achieve the recommended amount of physical activity, as per the guidelines, it would be necessary to increase the MVPA time as well as reduce the duration of sedentary behavior.

This study has a number strengths. The survey of 21 schools across multiple regions in Japan allowed us to more effectively probe the associations between physical activity and sleep habits. Even though the physical activity guidelines have been proposed [11–13], many children, in fact, do not meet these standards [36, 37]. Additionally, since the amount of physical activity declines after the child passes beyond the adolescent years [38], there is an urgent need to spread awareness regarding the physical activity guidelines. In this context, the present study elucidated the relationship between achieving guideline-recommended physical activity levels and sleep habits, providing knowledge and insights to support and promote children’s health by raising awareness of these guidelines.

This study has some limitations. First, as this was a cross-sectional study attempting to elucidate the sleep habits in relation to achieving guideline-recommended physical activity levels, it was not possible to establish causality. The time spent on light physical activity and participation in guided sports activities were not considered. Also, duration of the sedentary bouts could not be evaluated directly. The socio-economic status of the participants and their parent or guardian was not taken into consideration. In addition, regarding physical activity and sleep habits, subjective assessments were conducted. Going forward, it would be desirable to conduct such evaluations and assessments from both subjective and objective perspectives and accumulate further evidence concerning guideline-recommended physical activity levels and sleep habits.

## Conclusions

The present study investigated the association of sleep habits and lifestyle factors with the achievement of guideline-recommended physical activity levels in Japanese children. We showed an inverse correlation between the achievement status of guideline-recommended physical activity levels and excessive daytime sleepiness in boys in the fifth and sixth grades in elementary school. Additionally, among students in the seventh and eighth grades of junior high school, this achievement was inversely correlated with a substantial amount of social jetlag and skipped breakfast in boys and with excessive sleep duration on the weekends and prolonged screen time in girls. However, no significant associations were found for elementary school girls in the fifth and sixth grades. Taken together, our results indicate that sleep habits related to the achievement of guideline-recommended physical activity levels may differ by school type and gender. The relationship between sleep habits, lifestyle factors, and physical activity warrants further investigation and should be considered in the context of the future development of physical activity guidelines.

## Supporting information

S1 Database (XLSX)
